# A Stenotrophomonas maltophilia TetR-Like Transcriptional Regulator Involved in Fatty Acid Metabolism Is Controlled by Quorum Sensing Signals

**DOI:** 10.1128/aem.00635-23

**Published:** 2023-06-05

**Authors:** Xavier Coves, Marc Bravo, Pol Huedo, Òscar Conchillo-Solé, Andromeda-Celeste Gómez, Anna Esteve-Codina, Marc Dabad, Marta Gut, Xavier Daura, Daniel Yero, Isidre Gibert

**Affiliations:** a Institut de Biotecnologia i de Biomedicina (IBB), Universitat Autònoma de Barcelona (UAB), Cerdanyola del Vallès, Spain; b Departament de Genètica i de Microbiologia, Universitat Autònoma de Barcelona (UAB), Cerdanyola del Vallès, Spain; c CNAG-CRG, Centre for Genomic Regulation (CRG), The Barcelona Institute of Science and Technology, Barcelona, Spain; d Universitat Pompeu Fabra (UPF), Barcelona, Spain; e Catalan Institution for Research and Advanced Studies (ICREA), Barcelona, Spain; f Centro de Investigación Biomédica en Red de Bioingeniería, Biomateriales y Nanomedicina, Instituto de Salud Carlos III, Cerdanyola del Vallès, Spain; University of Tennessee at Knoxville

**Keywords:** quorum sensing, *N*-acyl homoserine lactones, DSF, fatty acid metabolism, TetR-like regulator, *Stenotrophomonas*

## Abstract

Stenotrophomonas maltophilia is an environmental bacterium as well as an emerging opportunistic multidrug-resistant pathogen. They use the endogenous diffusible signal factor (DSF) quorum sensing (QS) system to coordinate population behavior and regulate virulence processes but can also respond to exogenous N-acyl-homoserine lactone (AHL) signals produced by neighboring bacteria. The effect of these QS signals on the global gene expression of this species remains, however, unknown. Whole-transcriptome sequencing analyses were performed for exponential cultures of S. maltophilia K279a treated with exogenous DSF or AHLs. Addition of DSF and AHLs signals resulted in changes in expression of at least 2-fold for 28 and 82 genes, respectively. Interestingly, 22 of these genes were found upregulated by both QS signals, 14 of which were shown to also be induced during the stationary phase. Gene functions regulated by all conditions included lipid and amino acid metabolism, stress response and signal transduction, nitrogen and iron metabolism, and adaptation to microoxic conditions. Among the common top upregulated QS core genes, a putative TetR-like regulator (locus tag SMLT2053) was selected for functional characterization. This regulator controls its own β-oxidation operon (*Smlt2053*-*Smlt2051*), and it is found to sense long-chain fatty acids (FAs), including the QS signal DSF. Gene knockout experiments reveal that operon *Smlt2053*-*Smlt2051* is involved in biofilm formation. Overall, our findings provide clues on the effect that QS signals have in S. maltophilia QS-related phenotypes and the transition from the exponential to the stationary phase and bacterial fitness under high-density growth.

**IMPORTANCE** The quorum sensing system in Stenotrophomonas maltophilia, in addition to coordinating the bacterial population, controls virulence-associated phenotypes, such as biofilm formation, motility, protease production, and antibiotic resistance mechanisms. Biofilm formation is frequently associated with the persistence and chronic nature of nosocomial infections. In addition, biofilms exhibit high resistance to antibiotics, making treatment of these infections extremely difficult. The importance of studying the metabolic and regulatory systems controlled by quorum sensing autoinducers will make it possible to discover new targets to control pathogenicity mechanisms in S. maltophilia.

## INTRODUCTION

To rapidly adapt to environmental changes, bacteria modulate complex social behaviors through physiological processes collectively known as quorum sensing (QS). QS is a type of cell-cell communication that allows individuals of the same species or different species to collaborate and compete through production, release, and response to extracellular signals known as autoinducers (AIs) (1). By sensing these AIs, bacteria detect and respond to cell density through gene regulation. As a result, QS enables bacterial populations to synchronize gene expression to coordinate biofilm formation, virulence, and antimicrobial production and resistance, among others (2–4).

In the ubiquitous Gram-negative bacterium Stenotrophomonas maltophilia, the main QS system described so far is mediated by the fatty acid (FA) signal *cis*-11-methyl-2-dodecenoic acid, also called diffusible signal factor (DSF). S. maltophilia has emerged over the last decades as an opportunistic pathogen, causing nosocomial infections in the immunocompromised population, and it is commonly isolated from the lungs of cystic fibrosis patients (5, 6). One of the main concerns about the members of this species is that they tend to be intrinsically resistant to multiple antibiotics, with specific isolates showing evidence of adaptive resistance (7, 8). In addition, they have the ability to attach to abiotic and biotic surfaces and colonize medical devices and epithelial tissues forming biofilms (9), and they are prone to acquiring virulence factors through horizontal gene transfer (5). The DSF-mediated QS system has been proven to control biofilm formation, motility, and production of virulence and resistance factors in S. maltophilia and other DSF-producing bacteria (10–14), and a link has been established between DSF and susceptibility to some antibiotic classes in clinical strains (15).

Signal molecules of the DSF family mediate intra- and interspecies signaling in these species through components encoded in the regulation of pathogenicity factors (*rpf*) cluster. In S. maltophilia, the *rpf* cluster consists of two contiguous but convergent operons, *rpfFB* and *rpfCG* (10). Unlike other species, the distinctive feature of the DSF system in S. maltophilia is the presence of two allelic *rpf* variants (namely, *rpf*-1 and *rpf*-2) (10, 11). Strains with the *rpf-1* variant are more abundant, and they produce detectable DSF levels under laboratory conditions, whereas strains with variant *rpf-2* do not. However, under certain circumstances, strains of the *rpf-2* variant produce DSF endogenously, for instance, when high concentrations of exogenous DSF are present in the microenvironment (11). The genetic and biochemical mechanisms of DSF signaling, including DSF turnover, have been extensively studied in several members of the *Xanthomonadaceae* family (16–21). Nonetheless, in S. maltophilia, little is known about the regulation of this QS system.

Beyond DSF, the most common QS system in Gram-negative bacteria is based on *N*-acyl-homoserine lactones (AHLs) (22). In S. maltophilia, to the best of our knowledge, no canonical AHL synthases have been described (23, 24), despite that AHL activity has been associated with members of the *Stenotrophomonas* genus (25, 26). However, S. maltophilia can respond to AHL signals through its LuxR solo SmoR, which recognizes *N*-(3-oxooctanoyl)-homoserine lactone (3OC_8_-HSL) and enhances bacterial motility (27). On the other hand, S. maltophilia AHL-degrading strains have been isolated from the microbiota of aquatic organisms (28, 29), which indicates that these organisms can detect AHL molecules in the environment before they are hydrolyzed. The possibility of externally inducing AHL degradation, a quorum quenching (QQ) activity, is attractive because it offers the possibility of modulating virulence of some pathogens by interfering with the QS in highly competitive niches, such as the rhizosphere or the lungs of cystic fibrosis (CF) patients (30–32).

Owing to the limited knowledge of the effect of exogenous AI molecules in S. maltophilia and the potential therapeutic use their inactivation may have, the global expression profiling of S. maltophilia exposed to QS signals that are typical in its most common natural niches is studied. To start deciphering this response, transcriptomic analyses were performed by transcriptome sequencing (RNA-seq), comparing the expression profiles in two cell density-based stressing conditions, (i) growth in the presence of exogenously added QS signals, and (ii) the stationary phase of growth. Subsequently, genes that could be relevant for the pathogenesis and persistence of these bacteria, as well as those that could regulate or be involved in signal turnover, were further investigated. A tetR-like regulator was found to sense fatty acids, including DSF, and to control beta-oxidation family genes that influence virulence-associated phenotypes and could contribute to the degradation of exogenous long-chain fatty acids at high population density. This work provides a comprehensive global picture of how S. maltophilia responds to signals emitted by neighboring bacteria and the impact on virulence and bacterial fitness and the strategies used to overcome these stressing situations.

## RESULTS

### Transcriptomic analysis of S. maltophilia under growth conditions affected by QS autoinducers.

**FIG 1:**
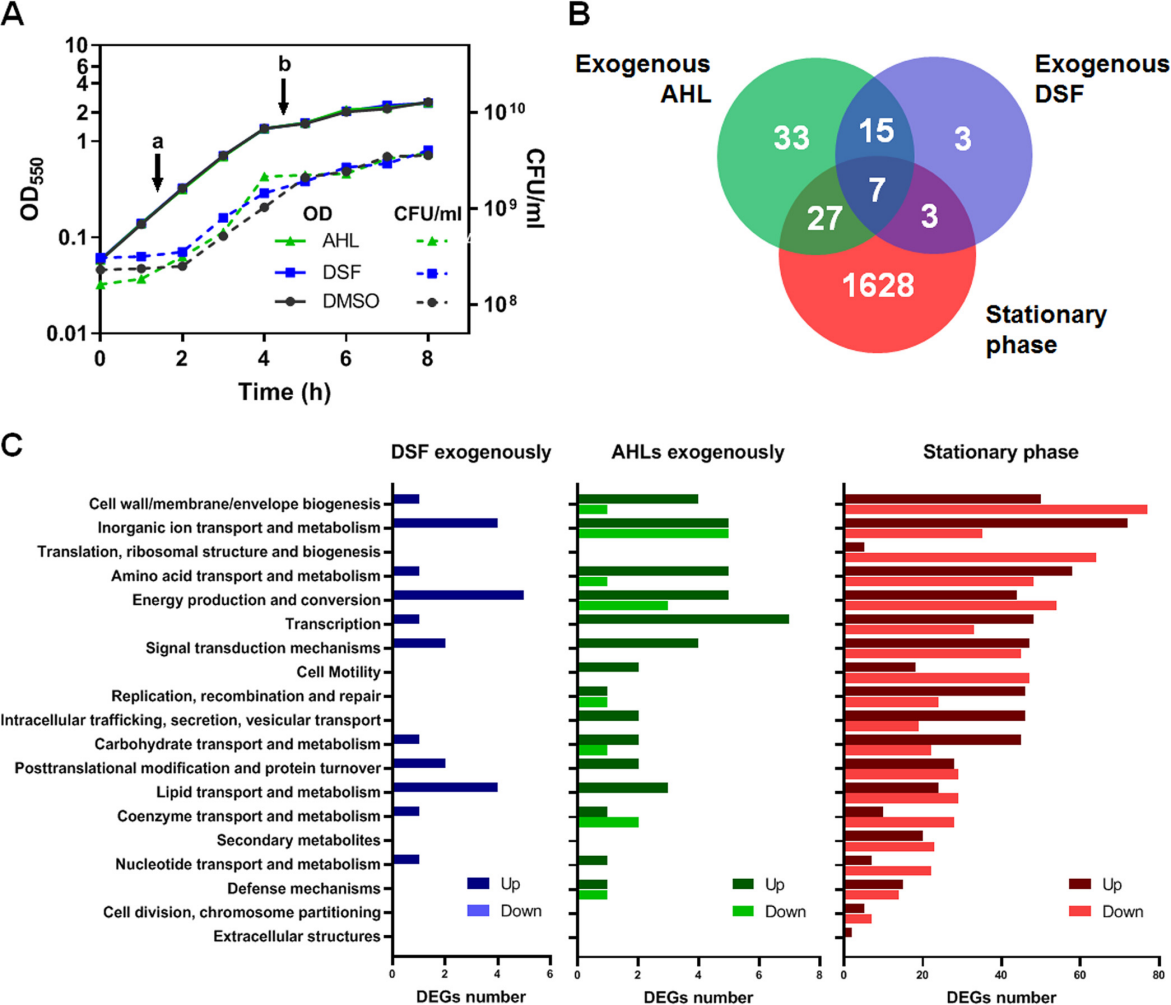
Experimental design and global analysis of differentially expressed genes (DEGs) under QS-inducing conditions. (A) Growth curves of the K279a strain under each condition, supplemented with AHLs, DSF, or DMSO as vehicle control. Cultures were induced at time point a, the start of the exponential phase, and harvested at point b, which corresponds to the mid- and late exponential phases of growth. In an independent experiment with a noninduced culture, samples were taken at time point b and at 24 h for comparisons with the stationary-phase transcriptome (see Fig. S1 in the supplemental material). (B) Venn diagram showing overlap of DEGs in response to the three assayed conditions. (C) Functional classification in clusters of orthologous groups (COG) of DEGs for each condition. COG categories are indicated in the *y* axis, while the *x* axis represents the number of DEGs matching each COG category at each condition. Genes with unknown function were not included in the graphs. Both the upregulated and downregulated genes are indicated under each condition with different color intensities. For the DSF condition, only upregulated genes were detected. The full list of significant DEGs is provided in the supplemental file 2.

To gain insight into the transcriptomic response of S. maltophilia K279a in the presence of QS signals, early exponential cultures were supplemented with either DSF or an AHL combo (C_8_-HSL, 3OC_8_-HSL, and C_10_-HSL, at 10 μM each), both dissolved in dimethyl sulfoxide (DMSO) (Fig. 1A, time point a) and samples collected 3 h after induction for RNA extractions (Fig. 1A, time point b). The used amounts of exogenously added AIs are in the range of concentrations required to exert a physiological effect (27, 33). Furthermore, during the time interval between the induction and the taking of samples for RNA-seq, there were no detectable amounts of the AIs in the culture supernatant of noninduced cultures. It is known that S. maltophilia does not produce AHLs at laboratory detection levels (24), and on the other hand, DSF production in S. maltophilia K279a starts at the beginning of the stationary phase between 10 and 15 h of culture (see Fig. S1 in the supplemental material).

**FIG 2:**
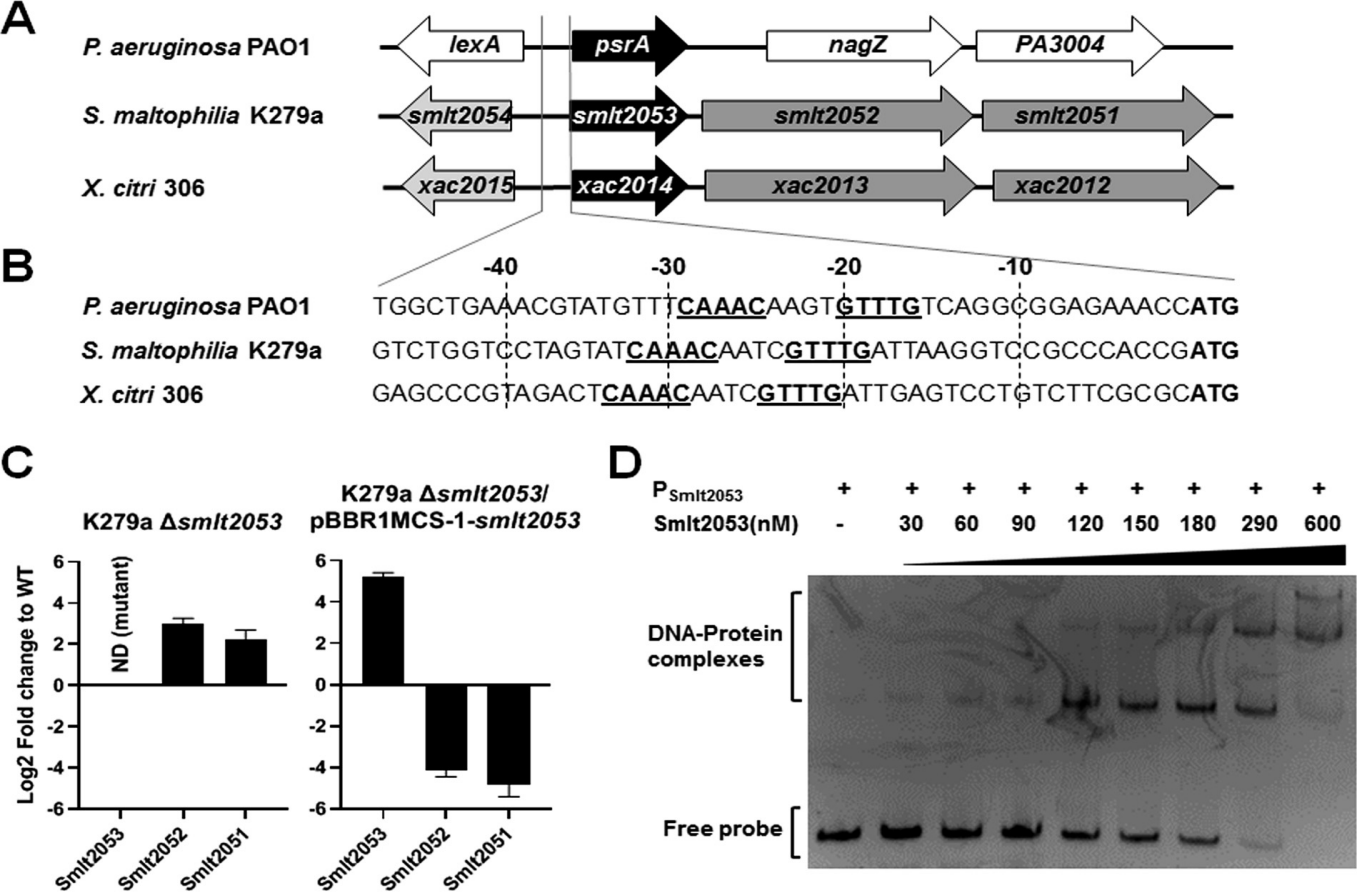
(A) Genomic context of orthologous TetR-like regulators in three *Gammaproteobacteria*. Gene names or locus tags are indicated in model genomes for strains PAO1 (GenBank accession no. NC_002516), K279a (GenBank accession no. NC_010943), and 306 (GenBank accession no. NC_003919). (B) DNA sequences of the operator regions of the three operons, with the likely DNA recognition box underlined. (C) Quantitative RT-PCR analysis of operon *Smlt2053*-*Smlt2051* gene expression in S. maltophilia K279aΔ*smlt2053* and its complemented variant. Fold changes are expressed relative to wild-type (WT) expression levels and normalized by the *rpoD* housekeeping gene. (D) Electrophoretic mobility shift assays for the promoter region of *Smlt2053* (P*_smlt2053_*) and the Smlt2053 protein. Molar protein concentrations are shown above the gel image, and 50 ng of the DNA probe was used.

This first transcriptomic analysis revealed a total of 82 or 28 differentially expressed genes (DEGs) after the treatment with AHLs or DSF, respectively, compared to the DMSO control group and, using as criterion for differential expression, a false-discovery rate (FDR)-adjusted *P* value lower than 0.05 and a fold change equal or greater than 2.0 (Fig. 1B and supplemental file 2). Upon AHL exposure, 58 genes were significantly upregulated, and 24 genes downregulated, whereas DSF had only an inducing effect. Clusters of orthologous groups (COG) analysis showed enrichment in the categories of energy production and inorganic ion transport and metabolism for DEGs after treatment with both AI molecules (Fig. 2C). DSF also upregulated genes related to lipid transport and metabolism, whereas AHLs induced amino acid and nitrogen metabolism. To develop a general picture of the biological processes affected by DSF and AHL signals, gene ontology (GO) enrichment analyses were performed on genes upregulated by both DSF and AHLs (Fig. S3 and S4). As with the COG analysis, oxidation-reduction and energetic processes were enriched in the common DEG data set.

Remarkably, for 22 functional genes, there was induction by both QS signals, representing 79% of DSF and 38% of AHL DEGs (Fig. 1B and Table 1). The fold regulation of six common induced genes was validated by quantitative real-time PCR (qRT-PCR), and the results closely mirrored the values obtained by RNA-seq (Fig. S2). In the common data set, nine of the overexpressed genes are part of four operons (Table 1). One of these transcriptional units is a putative β-oxidation operon in the K279a genome (*Smlt0264-Smlt0268*), and the other is directly involved in iron storage (*Smlt3600-Smlt3601*). Top upregulated genes (>5-fold changes) in both conditions encode a TetR family regulator (Smlt2053) and the protein phenylalanine 4-monooxygenase (Smlt0077).

**TABLE 1:** Common genes induced by both AHLs and DSF signals compared to the expression profile during the stationary phase of growth

K279a locus tag^*a*^	Fold expression^*b*^			Protein function (gene name)
	DSF	AHLs	STAT	
Smlt0076^*c*^	2.82	2.14	2.99	Imm26 family immunity protein
Smlt0077^*c*^	7.77	7.17	3.55	Phenylalanine-4-monooxygenase (*phhA*)
Smlt0264^*c*^	3.54	2.72	2.04	CoA-acylating methylmalonate-semialdehyde dehydrogenase
Smlt0265^*c*^	2.71	2.73	1.96	Acyl-CoA dehydrogenase
Smlt0266^*c*^	2.60	2.14	2.17	Enoyl-CoA hydratase
Smlt1574	4.12	2.84	2.41	Bacteriohemerythrin
Smlt1687^*c*^	2.89	3.84	1.47	4Fe-4S binding protein
Smlt1688^*c*^	2.35	2.03	2.78	Hypothetical protein
Smlt1690	4.73	3.93	4.45	Oxygen-independent coproporphyrinogen III oxidase (*hemN*)
Smlt1757	3.24	3.51	1.34	C-type cytochrome
Smlt1758	2.65	3.51	1.55	Cytochrome^*c*^
Smlt2053	6.52	7.33	5.23	TetR/AcrR family transcriptional regulator
Smlt2137	2.05	2.51	3.50	Universal stress protein
Smlt2180	2.70	2.62	18.26	Glycoside hydrolase family 92 protein
Smlt2775	3.79	2.81	2.9	NarK family nitrate/nitrite MFS transporter
Smlt2944	4.88	4.29	−1.05	DcaP family trimeric outer membrane transporter
Smlt3600^*c*^	2.98	3.13	1.52	Peroxiredoxin
Smlt3601^*c*^	3.11	2.66	2.01	Ferritin-like domain-containing protein
Smlt3647	3.05	2.68	2.00	Alpha-/beta-hydrolase
Smlt3805	2.79	2.21	−1.18	OMP beta-barrel
Smlt4268	2.03	2.40	1.65	Bifunctional isocitrate dehydrogenase kinase/phosphatase (*aceK*)
Smlt4591	5.33	4.03	3.26	Universal stress protein

a Genes are organized according to their locus tags.

b Fold expression in comparison to the control. STAT, stationary phase (compared to the logarithmic state in untreated cultures).

c Gene is part of an operon.

### QS signal-induced genes are also deregulated in stationary-phase cells of S. maltophilia.

To investigate whether the inducing effect of exogenous QS signals during exponential cultures occurs also during natural high-cell-density situations, we analyzed the global gene expression of untreated K279a cells in the stationary phase (24 h; optical density at 550 nm [OD_550_], ~5.0) compared with the mid- and late sexponential phase (5 h; OD_550_, 1.5) (Fig. S1). S. maltophilia is considered a fast-growing organism, and strains usually reach the stationary phase of growth in 20 to 24 h with a high cell density (34, 35). Differential expression analysis of the RNA-seq samples collected at both phases showed a total of 1,673 DEGs, representing 37% of total genomic content of S. maltophilia K279a. Of those genes, 908 were, under the mentioned criterion, upregulated, whereas 765 were found downregulated (the full list of DEGs is provided in the supplemental file 2). When all DEGs were analyzed in terms of their functional categories (Fig. 1C), main differences were observed in COG categories related to metabolism and regulation of gene expression. For instance, “inorganic ion transport” and “amino acid metabolism” were the most enriched categories in the upregulated data set. On the contrary, the fraction of genes assigned to “energy production” and “translation and ribosomal structure” represented the most enriched categories in the downregulated data set. The category “cell wall biogenesis” was highly enriched in both groups.

Most of the top upregulated genes (>5-fold changes) belong to energetic metabolism, including genes involved in oxidation-reduction processes (e.g., the *cta* operon encoding the cytochrome *c* oxidase and putative cytochrome *bd* subunit genes), DNA replication (putative *nrd* operon *Smlt2839*-*Smlt2842*), metabolite transport, and secretion. The major extracellular protease StmPR1 (*Smlt0686*), the glycoside hydrolase *Smlt2180*, and the quaternary ammonium compound efflux transporter *Smlt2852* are among the most overexpressed genes in the stationary phase. A variety of transcriptional regulators and sigma factor family genes were also overexpressed in the stationary phase, including the uncharacterized regulators Smlt2428 and Smlt2711 and the TetR family regulator Smlt2053. Particularly, the sigma-70 family RNA polymerase factor Smlt1750 (13.4-fold) could control stress response genes at the beginning of the stationary phase. Among the downregulated genes stand out members of the cell division cluster Smlt0747-Smlt0760 (*mur* and *fts* genes), the transcriptional repressor *lexA* and recombinase *recA*, two flagellum-associated operons, pilin-associated and chemotaxis genes, and the Ax21 family proteins Smlt0387 and Smlt0184.

Most of the DEGs after the treatment with DSF or AHLs were also found differentially expressed in the stationary phase of growth (64% of DSF and 51% of AHL DEGs) (Fig. 1B), which could indicate that the addition of QS AIs exogenously affects metabolic pathways in the same way as entering the stationary phase. For instance, the most downregulated genes, after adding a cocktail of AHLs, were also significantly repressed in the stationary phase. These genes belong to the *nar* operon (*Smlt2769*-*Smlt2774*), which encodes the respiratory nitrate reductase for nitrate respiration under oxygen-limiting conditions and includes the nitrate/nitrite transporter gene *narK*. On the contrary, an additional *narK* gene (*Smlt2775*), located immediately upstream to the *nar* operon, was found upregulated in the three conditions assayed. Interestingly, 14 out of these 22 genes commonly induced by both QS molecules (Table 1) were also found upregulated in the stationary phase, including the TetR family regulator *Smlt2053*.

### Smlt2053 is a transcriptional regulator that autoregulates its own operon.

Among the common upregulated QS core genes (i.e., the genes upregulated under all conditions related to QS activation or sensing), we focused on the regulatory gene *Smlt2053*, as it was the first and the second most upregulated gene in the AHLs and DSF setups, respectively. In addition, this gene was also upregulated in the stationary phase of growth, with a fold increase of 5.23 (Table 1). It is annotated as a putative TetR and AcrR regulator; therefore, we initially hypothesized that it could act as a cytoplasmic regulator of the QS cascade or as a regulator of the quorum quenching pathway.

We initially performed sequence comparisons to identify homologues among related bacteria and obtain clues on their biological function. The closest regulatory protein with known function was identified in Xanthomonas citri (Xac2014), sharing 89% amino acid identity. Besides *Xanthomonadaceae* proteins, Smlt2053 also shares 39% identity with PsrA (PA3006) from Pseudomonas aeruginosa PAO1. Reciprocal BLAST confirmed that Smlt2053 is likely an ortholog of the P. aeruginosa protein PsrA. While *psrA* is organized as a single gene in the P. aeruginosa genome, the genomic organization of *Xac2014* and *Smlt2053* is identical, with both genes conforming an operon encoding an additional 3-hydroxyacyl-CoA dehydrogenase (*Xac2013*/*Smlt2052*) and acetyl-CoA C-acyltransferase (also known as 3-ketoacyl-CoA thiolase) (*Xac2012*/*Smlt2051*) (Fig. 2A), variants of the two enzymes of fatty acid catabolism FadB and FadA, respectively (36). The homology found between the three transcription factors is mainly restricted to the N terminus, which corresponds to the DNA binding domain (37). In addition, the DNA recognition sequence of PsrA in P. aeruginosa has been identified as G/CAAAC(N_2-4_)GTTTG/C (Fig. 2B) (38). Notably, both *Smlt2053* and *Xac2014* present an identical DNA recognition sequence in their operator region, and their position with respect to the +1 is very similar. The similar N-terminal sequence of the three proteins, as well as the identical DNA recognition sites found in their operator sequences, suggests that the three transcription factors bind to their operator sequences, repressing their own expression, as reported for PsrA (38–40).

We investigated the autoregulatory activity of Smlt2053, as well as the role that may exert on other genes, using different approaches. First, a series of qRT-PCRs were conducted in an S. maltophilia K279a deletion mutant for gene *Smlt2053* (K279aΔ*smlt2053*) and compared with the wild-type (WT) strain. For mutant construction, a markerless gene deletion was obtained without affecting its own promoter to avoid the polar effect on genes *Smlt2052* and *Smlt2051* in the same operon. Genes forming an operon with *Smlt2053* were selected for this analysis. The absence of *Smlt2053* resulted in a significantly increased expression of the adjacent genes *Smlt2052* and *Smlt2051* compared to the wild-type background, strongly suggesting its autorepression activity and thus validating the operon organization of *Smlt2053*-*Smlt2051* (Fig. 2C). Genetic complementation of mutant strain K279aΔ*smlt2053* using a multicopy plasmid restores operon repression to levels higher than in the wild-type strain (Fig. 2C). To confirm the autoregulation of *Smlt2053*, we tested the direct interaction between protein Smlt2053 and its own promoter region *in vitro* using electrophoretic mobility shift assay (EMSA) (Fig. 2D). The EMSA results showed that the protein Smlt2053 specifically binds to a DNA fragment containing the promoter region, therefore indicating the binding of the regulator protein to its own promoter. The experiment revealed that as the concentration of the protein increases, an additional electrophoretically distinct protein-DNA complex with high affinity is formed. These findings suggest a different genomic target for the transcriptional regulator in the promoter P*_Smlt2053_*. Careful examination of the promoter region revealed an additional inverted repeat which resembles the consensus motif G/CAAAC(N_2-4_)GTTTG/C (Fig. S5A). Both consensus sequences are conserved among genotypically different S. maltophilia strains (Fig. S5B) as well as the operon genes. On the other hand, the protein Smlt2053 did not bind to the promoter region of other genes used as control, which demonstrates its specificity (Fig. S6).

To further investigate the role of Smlt2053 in QS and global regulation in S. maltophilia, additional transcriptomic experiments were performed on exponential cultures of the mutant K279aΔ*smlt2053* and the isogenic parent strain K279a and compared. RNA-seq data showed seven genes whose expression levels differed by at least 2-fold from those in the wild-type strain (*P*_adj_ < 0.05) (supplemental file 2). Five genes were downregulated in the mutant strain, while the adjacent genes *Smlt2052* and *Smlt2051* were upregulated, hence confirming the autorepression activity observed by qRT-PCR for this operon (Fig. 2C). However, the results for the other five genes could not be validated by qRT-PCR using independent samples. Additionally, an inverted repeat sequence similar to that found in the promoter of *Smlt2053* was not found in any of the promoter regions of these five genes.

### Fatty acids, including DSF, affect the binding of Smlt2053 to its promoter.

**FIG 3:**
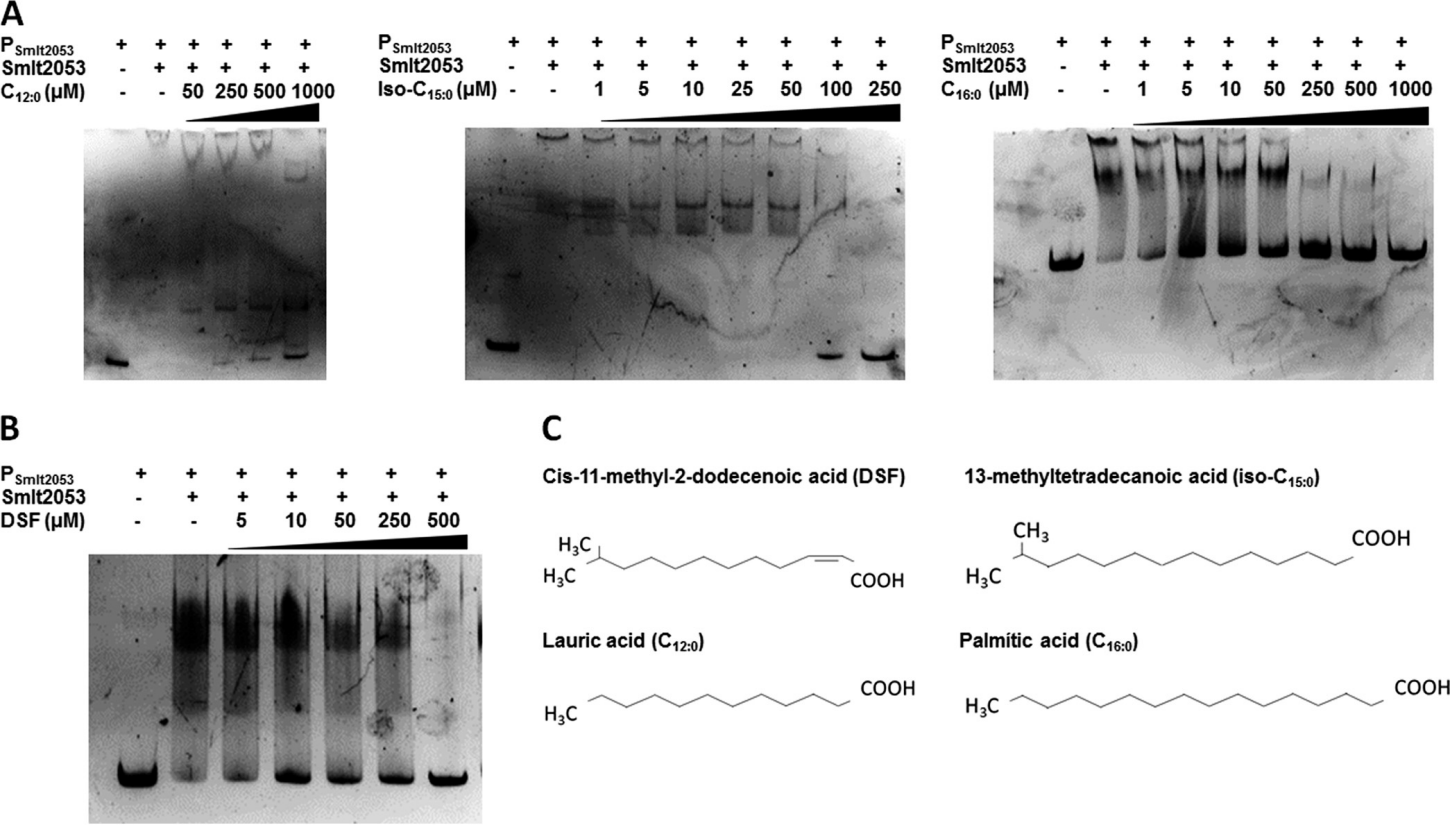
EMSA of Smlt2053 protein with the DNA probe P*_smlt2053_* in the presence of exogenous fatty acids. After 30 min of protein-DNA incubation, increasing amounts of each fatty acid were added to the reaction mixture for an additional 30 min. The protein Smlt2053 was used at a fixed concentration of 429 nM (11.25 ng/μL). (A) EMSAs in the presence of different fatty acids as follows: lauric acid (C_12:0_) (left), 13-methyltetradecanoic acid (iso-C_15:0_) (center), and palmitic acid (C_16:0_) (right). Lane 1 in all three experiments included only the DNA probe (P*_smlt2053_*). Lane 2 included the protein and the vehicle used to dissolve each fatty acid as follows: water containing 1% Brij 58 was used for C_12:0_ and iso-C_15:0_, whereas 10% methanol was used for C_16:0_. (B) DSF at concentrations ranging from 5 to 500 μM was added to the reaction. Lane 1 contained neither protein nor DSF and was used as a probe control. Lane 2 included protein and the same volume of DMSO (solvent) used at the highest concentration as a vehicle control. In the right panel, the structure of each fatty acid used in this figure is indicated to highlight similarities between them. (C) Structure of DSF and long-chain fatty acids used in EMSA.

Since Smlt2053 is the ortholog to P. aeruginosa PsrA, a known TetR regulator of the β-oxidation pathway that binds fatty acids (41), we hypothesized that Smlt2053 would behave in a similar way. We used EMSA to monitor the effect of short-, medium-, and long-chain fatty acids on DNA-*Smlt2053* binding (Fig. 3A and Fig. S7). The presence in the mixture of a short (C_4_)- and medium (C_8_)-length fatty acid did not affect the binding of the Smlt2053 protein to the probe containing its own regulatory region (Fig. S7). Medium (C_8_ and C_12_)-length fatty acids partially affected this binding, but only at millimolar concentrations. According to the EMSA results, when long-chain fatty acids containing more than 12 carbons in its backbone were included in the binding reaction, with almost all the DNA probe P*_smlt2053_* bound to the Smlt2053 protein, an increasing amount of free probe was observed, correlating with the increasing amounts of each fatty acid (Fig. 3A). This confirms the ability of Smlt2053 to detect and bind long-chain fatty acids, leading to the activation of at least part of the β-oxidation machinery. In addition, 13-methyltetradecanoic acid (iso-C_15:0_), the most abundant fatty acid in S. maltophilia, which stimulates DSF synthesis (11), also reverted the *Smlt2053*-DNA binding (Fig. 3A).

Considering the lipid nature of DSF and its connection with iso-C_15:0_, together with its participation in the regulation of *Smlt2053* expression, we next examined if DSF could also act as a ligand for this regulator. Increasing amounts of DSF disrupted the shift observed between *Smlt2053* and its own promoter region P*_smlt2053_*, indicating that *Smlt2053* responds to the main QS signal of S. maltophilia (Fig. 3B). These results suggest that *Smlt2053* participates in the control of the catabolism of medium- and long-chain fatty acids, including QS signal turnover.

### Smlt2053 participates in biofilm formation in S. maltophilia.

**FIG 4:**
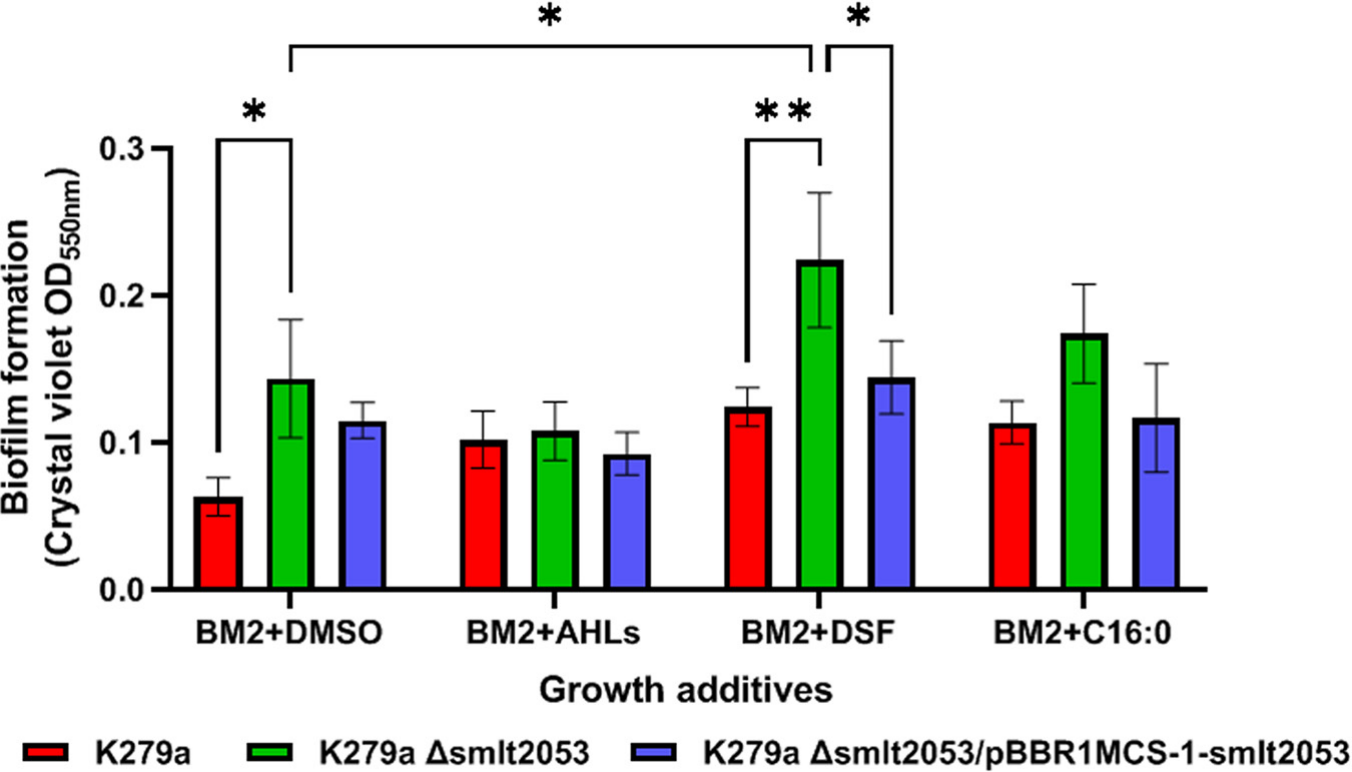
Effect of mutation in *Smlt2053* on S. maltophilia K279a biofilm growth under static conditions in the presence of QS autoinducers and fatty acids. Cells were grown in a polystyrene microtiter plate in modified BM2 minimal media at 37°C and supplemented with DSF, AHL, or sodium palmitate (C_16:0_) at 10 μM each or with DMSO as control. The samples were assayed in triplicate, and the error bars represent standard deviation. Tukey's multiple-comparison test (two-way ANOVA) was used to determine the significance of the data between groups (*, *P < *0.05; **, *P < *0.01).

To study the influence of the operon regulated by the TetR-like transcription factor Smlt2053 in bacterial phenotypes, biofilm formation in the mutant strain K279aΔ*smlt2053* was first evaluated. It is known that the ability of S. maltophilia to form biofilm is regulated in part by its QS system (10, 11, 15). Biofilm formation in a BM2 medium with glucose and Casamino Acids as carbon and nitrogen sources, respectively, showed that the lack of the regulator *Smlt2053* increases the ability of cells to form biofilm (Fig. 4). The experiment also demonstrated that supplementing the cultures with DSF increased the observed differences between the mutant and wild type. These phenotypes could be reverted by complementation with the cloned gene inserted in a replicative plasmid. None of the strains tested showed significant differences in terms of growth or DSF production in the assayed conditions (Fig. S8 and S9).

Finally, the role that the operon *Smlt2053*-*Smlt2051* may play in the metabolism of fatty acids was studied. A mutant strain for the whole operon (K279aΔ*smlt2053-2051*), which includes *fadB* and *fadA* gene variants, was also generated. The deletion of the components of this β-oxidation operon does not seem to alter the global fatty acid metabolism and regulation in S. maltophilia as determined by the fatty acid profile of K279a WT and mutants K279aΔ*smlt2053* and K279aΔ*smlt2053-2051* (Table 2). Fatty acid profiles with predominance of the 13-methyl-tetradecanoic acid (*iso*-15:0) are characteristic of the genus *Stenotrophomonas*, and no significant differences were observed in the abundance of all the molecules detected among the three strains analyzed. These results could indicate that the enzymes of the operon *Smlt2053*-*Smlt2051* are not part of the main fatty acid catabolism pathway in S. maltophilia or that they are simply redundant (there exist other enzymes with 3-hydroxyacyl-CoA dehydrogenase and 3-ketoacyl-CoA thiolase activities in the genome).

**TABLE 2:** Total cellular fatty acids in S. maltophilia K279a and mutant strains K279aΔ*smlt2053* and K279aΔ*smlt2053-2051*

Fatty acid	Percentage in K279a	Percentage in K279aΔ*smlt2053*	Percentage in K279aΔ*smlt2053-2051*
10:0	0.43	0.30	0.40
11:0 iso	2.31	2.45	2.56
11:0 iso 3OH	1.73	1.58	1.83
13:0 iso	0.33	0.33	0.39
12:0 iso 3OH	0.30	0.31	0.27
12:0 2OH	0.51	0.48	0.45
12:0 3OH	1.96	1.84	2.18
14:0 iso	1.50	1.54	1.10
14:0	2.82	3.06	2.99
13:0 iso 3OH	2.91	2.86	3.27
13:0 2OH	0.46	0.53	0.51
15:1 iso F	0.89	0.89	0.92
15:0 iso	38.77	39.06	38.13
15:0 anteiso	13.31	14.83	13.60
16:0 iso	2.71	2.55	1.97
16:1 w9c	1.57	1.55	1.60
16:0	12.23	12.11	12.45
17:0 iso	4.43	3.96	4.32
17:0 anteiso	0.36	0.35	0.37
18:1 w9c	0.80	0.72	0.81
18:0	0.58	0.56	0.63

## DISCUSSION

Bacteria are commonly found in complex and heterogeneous communities. Within these environments, the exchange of signals governs the interactions and behaviors of the microbial populations. Typically, these functions involve both competitive and cooperative behaviors, such as production of virulence factors and intra- and interspecies communication (3, 42). The QS system in S. maltophilia is based on the fatty acid DSF, but it can also respond to exogenous AHLs (24). However, the effect of exogenous QS signals has not been studied in detail in this species. To this end, we supplemented early-log-phase cultures of the model strain K279a with either DSF or AHLs at concentrations that have a physiological effect and subjected each condition to RNA-seq. Between 50% and 65% of the DEGs, after treatment with each of the AIs in the mid- and late exponential phases (5 h; OD_550_, 1.5), are found also deregulated in noninduced cells collected in the stationary phase. These include genes required for energy generation pathways and stress response under oxygen-limiting conditions, β-oxidation, and iron storage, among others. It has been shown in other bacteria that QS regulates the transition into the stationary phase in tight connection with starvation sensing (43), preparing the cells for a critical situation of stress due to lack of nutrients (44). The simple comparison of stationary-phase versus logarithmic-phase transcriptomes, done in parallel, highlights the stressing environment of a high-density culture of S. maltophilia, which implies the activation of multiple stress pathways due to nutrient limitation and accumulation of metabolites, as seen in other bacteria (43, 45).

Among the most enriched COG categories found in both DSF and AHL conditions, those related to energy conversion are overrepresented, particularly those involving lipid and amino acid metabolism. This result suggests that QS signals are primarily metabolized by S. maltophilia and are probably used as an energy source in complex and diverse communities. Remarkably, an overlap of genes whose expression was regulated by both DSF and AHLs was observed. These findings agree with previous studies in the unrelated Burkholderia cepacia complex and Burkholderia cenocepacia species, where the DSF and AHL stimuli overlap through modulation of the intracellular levels of c-di-GMP (46, 47). QS systems in both species control, among others, genes related to iron metabolism or proteins that use iron as a cofactor, as well as regulators of the stress response and general metabolism. Likewise, genes that fall in the same categories were found to be differentially expressed under the conditions presented here. The small amount of DEGs after induction with DSF relative to other conditions deserves to be discussed, considering that this AI is sensed by the main QS system in S. maltophilia. The natural DSF production in these bacteria corresponds with a high-density culture just at the entrance of the stationary phase as it occurs with other members of the *Xanthomonadaceae* family (14). At the beginning of the stationary phase, some nutrients begin to be scarce, and this nutritional stress influences the expression of many other genes that have not been directly deregulated by the addition of DSF exogenously in our experimental design. On the other hand, supplementing the cultures with DSF also did not impact the regulation of the *rpf* cluster, at least in the *rpfF*-1 variant strain K279a. In Xanthomonas campestris pv. *campestris*, DSF production reaches a peak in the early stationary phase, and the level subsequently declines; however, transcription of *rpfF* is detected throughout the growth curve, with a marked increase at the entry of the stationary phase (48). Also, addition of DSF during exponential growth did not affect the subsequent expression of *rpf* genes in X. campestris pv. *campestris*, indicating that DSF does not autoregulate its own synthesis, at least at the transcriptional level (48).

Among the DEGs that were found upregulated in all tested conditions, we selected the transcriptional regulator of the TetR/AcrR family (*Smlt2053*) for functional characterization. EMSA and transcriptomic analysis showed that *Smlt2053* can bind to a DNA fragment containing the proposed consensus site found in its own regulatory region and regulates the expression of its own operon through long-chain fatty acid sensing. Basically, Smlt2053 binds to its target regulated genes, repressing their expression at low intracellular fatty acid concentration. Furthermore, the operon *Smlt2053*-*Smlt2051* is part of the core genome of the species S. maltophilia, and its promoter sequence is conserved among genotypically diverse strains. In P. aeruginosa, the orthologous protein PsrA was found to recognize long-chain fatty acids through the C terminus and derepress its own expression upon the presence of the ligand (49, 50). Moreover, PsrA activates RpoS, the stationary-phase sigma factor, and it also regulates the expression of LexA (38, 40). Contrary to our results in S. maltophilia, transcriptome analysis of the Δ*psrA* mutant in P. aeruginosa showed a broad network of genes whose expression is modulated by PsrA (41). This work demonstrated the involvement of PsrA in the regulation of β-oxidative enzymes, as shown here for Smlt2053. More specifically, the authors proposed a role for this regulator in sensing free fatty acids from the environment to derepress a β-oxidation operon to degrade long-chain fatty acids.

The closest ortholog of Smlt2053 in the related species Xanthomonas citri, the TetR regulator XAC2014, was found to be the target of TfmR (type 3 secretion system [T3SS] and fatty acid mechanism regulator), also supporting its role in the fatty acid pathways and virulence (36). *Smlt2053* and *XAC2014* harbor a palindromic DNA binding motif in the upstream promoter region, which is almost identical to the one characterized in P. aeruginosa [G/CAAAC(N_2-4_)GTTTG/C]. All of these results, together with the fact that they both form an operon with two genes coding for enzyme variants of the β-oxidation pathway, suggest a similar role in lipid catabolism in the *Xanthomonadales* family. Interestingly, DSF showed the same effect as long-chain fatty acids as an inducer of repressor *Smlt2053*. This result suggests that Smlt2053 could act as an intracellular receptor of DSF, acting as a key regulator of the signal turnover, by leading the excess of FAs to the β-oxidation pathway together with RpfB (Acyl-CoA ligase) and enzymes from operon *Smlt0264*-*Smlt0268*. RpfB is involved in DSF processing in X. campestris pv. *campestris* and also counteracts the nonspecific thioesterase activity of RpfF (51). An enzymatic breakdown of DSF within the cell has already been suggested by others after noticing that DSF concentration declines as the stationary phase progresses (48). Along with its role in fatty acid metabolism, both TfmR in X. citri and PsrA in P. aeruginosa are involved in the metabolic control of virulence-associated phenotypes (36, 52). A P. aeruginosa *psrA* mutant is defective in biofilm formation, which is contrary to what happens with the mutant K279aΔ*smlt2053* in S. maltophilia. No gene directly related to biofilm formation seems to be regulated by Smlt2053 in S. maltophilia, and the effect could be thus indirect.

The different studies of global gene expression presented here have also yielded other interesting data. For instance, the gene coding for the oxygen-binding protein bacteriohemerythrin was found upregulated in the stationary phase and after the induction with both DSF and AHLs. The ortholog of S. maltophilia bacteriohemerythrin in P. aeruginosa plays a role in growth under microoxic conditions, particularly in a mutant of the QS regulator LasR (53). Adaptation of bacterial cells to the micro-oxic environment is important at the stationary phase where oxygen concentration dissolved in the growth medium is low due to the high cell density. The role played by nitrate during the stationary phase of growth is also important. The nitrate reduction operon *narGHJIK1* was found downregulated in the stationary phase, and an additional *narK* gene (*Smlt2775*) activates its expression at this stage and after adding the QS AIs. *Smlt2775* is orthologous to *narK2* in Pseudomonas aeruginosa, which is exclusively required as a nitrate/nitrite transporter under denitrifying conditions (54). Contrary to P. aeruginosa, our results indicate that the transcription of *narK2* in S. maltophilia could be independent of the operon *narGHJIK1*. In enterobacteria, the synthesis of different nitrate reductases takes place during different phases of bacterial growth in response to variations in carbon source needed to maintain aerobic respiration (55).

This work, in addition to providing clues about the regulation mechanism of QS in S. maltophilia, paves the way to finding molecules that inhibit QS-dependent virulence factors. The QS system seems to confer a fitness advantage in high-density growth conditions, such as biofilm formation, and this could be modulated through quorum quenching (QQ). In particular, S. maltophilia isolates have shown QQ activity against AHLs. There is no report of DSF QQ activity in S. maltophilia, although it has been observed in other species (24, 25, 56). Fatty acids are now recognized as a potential alternative to conventional antibiotics because they have shown antibiofilm and antivirulence activity (57). It is known that one of the main mechanisms of action of candidate lipidic molecules in the cell is QQ; therefore, a therapeutic intervention that modulates lipid-mediated QS pathways in S. maltophilia could be an unconventional alternative to currently available antimicrobials.

## MATERIALS AND METHODS

### Bacterial strains and reagents.

All bacterial strains used in this study are listed in Table 3. S. maltophilia K279a (58) was used as the reference strain in this study and Xanthomonas campestris pv. *campestris* 8523 pL6engGUS as the DSF reporter strain (59). Synthetic QS signals [*N*-octanoyl-HSL (C_8_-HSL), *N*-(3-oxooctanoyl)-HSL (3OC_8_-HSL), *N*-decanoyl-HSL (C_10_), and *cis*-11-methyl-2-dodecenoic acid (DSF)] were obtained from Sigma-Aldrich. Stocks were prepared in DMSO at a concentration of 20 mg/mL, aliquoted, and stored at −20°C until use. Free fatty acids used in this study were purchased from Sigma-Aldrich in the sodium salt form. Sodium butyrate and sodium octanoate were dissolved in water, sodium dodecanoate and 13-methyltetradecanoic acid (iso-C_15:0_) were dissolved in water containing 1% Brij 58, and sodium palmitate (C_16:0_) was dissolved in 10% prewarmed methanol.

**TABLE 3:** Bacterial strains and plasmids used in this work

Strain or plasmid	Genotype and/or relevant characteristics	Source and/or reference
Strains		
Stenotrophomonas maltophilia		
K279a (wild type)	Clinical isolate and the genetic reference strain	Laboratory collection, 58
K279aΔ*smlt2053*	K279a carrying a deletion in gene *Smlt2053*	This study
K279aΔ*smlt2053-2051*	K279a carrying a deletion in operon *Smlt2053*-*Smlt2051*	This study
Xanthomonas campestris pv. *campestris*		
8523 pL6engGUS	*rpfF* mutant, plasmid pLAFR6 carrying *engXCA*:*gusA* fusion. DSF reporter strain	59
Escherichia coli		
DH5α	F^−^ Φ80*lacZ*ΔM15 Δ(*lacZYA-argF*) U169 *recA1 endA1 hsdR17*(r_K_^−^ m_K_^+^) *phoA supE44 thi-1 gyrA96 relA1*λ^−^	Laboratory collection, 70
SY327	Δ(*lac pro*) *argE*(*Am*) *recA56 rif ^r^ nalA* λ *pir*	71
BL21(DE3)	F^–^ *ompT gal dcm lon hsdS_B_*(*r_B_*^–^ *m_B_*^–^) λ(DE3 [*lacI lacUV5*-*T7p07 ind1 sam7 nin5*]) [*malB*^+^]_K-12_(λ^s^)	Novagen
Plasmids		
pGPI-SceI-XCm	Mobilizable suicide vector; carries the R6Kγ origin of replication, the I-SceI recognition site, and a *xylE* reporter gene, Cm^r^, Tp^r^	67
pΔ*smlt2053*-US’DS’	pGPI-SceI-XCm containing the upstream and downstream flanking DNA regions of the *smlt2053* gene	This study
pΔ*smlt2053-2051*-US’DS’	pGPI-SceI-XCm containing the upstream and downstream flanking DNA regions of the *smlt2053*-*smlt2051* operon	This study
pRK2013	RK2-derived helper plasmid carrying the *tra* and *mob* genes for mobilization of plasmids containing *oriT*, Kan^r^	72
pDAI-SceI-SacB	Mobilizable broad host range plasmid; carries the gene for the I-SceI homing endonuclease and the *sacB* gene, Tet^r^	67, 73
pBBR1MCS-1	Broad-host-range cloning vector used for complementation, low copy, Cm^r^	74
pBBR1MCS-1-*smlt2053*	pBBR1MCS-1 with the gene *Smlt2053* inserted between sites XbaI and HindIII, Cm^r^	This study
pET28a-TEV	Bacterial expression vector with T7-*lacO* promoter, hexa His tag, TEV cleavage site, Kan^r^	75
pET28a-Smlt2053	Plasmid for Smlt2053 protein production in E. coli	This study

### Growth conditions and growth curves.

Unless otherwise stated, K279a and derived strains were routinely grown at 37°C in lysogeny broth (LB) medium on a rotatory shaker at 200 rpm supplemented with the appropriate antibiotic when necessary. For phenotypic analysis, strains were grown in the defined medium BM2-glucose minimal medium (62 mM potassium phosphate buffer, pH 7.0, 2 mM MgSO_4_, 10 μM FeSO_4_, and 0.4% glucose) supplemented with 0.5% Casamino Acids (10). For growth curve experiments, fresh colonies from each strain were picked from agar plates and incubated overnight in 10 mL of the corresponding culture broth at 37°C with shaking. Overnight cultures were diluted to an optical density at 550 nm (OD_550_) of 0.05, and bacterial suspensions were used to inoculate a conventional 96-well microtiter plate (triplicate samples). The plates were incubated inside a Multiskan FC microplate photometer (Thermo Fisher Scientific) under constant circular shaking at 37°C for 24 h for bacterial growth, and the OD_550_ was measured every 15 min. The X. campestris pv. *campestris* reporter strain was routinely grown in NYG medium (0.5% peptone, 0.3% yeast extract, and 2% glycerol) supplemented with tetracycline at 10 μg/mL at 28°C.

### Culture conditions and RNA extractions for RNA-seq.

Two separate RNA-seq experiments were performed, (i) comparison of the changes in global gene expression in S. maltophilia K279a under QS-inducing conditions, and (ii) transcriptome analysis of K279a mutant strain with a deletion of the *Smlt2053* gene (K279aΔ*smlt2053*). All experiments were performed with triplicate samples. For the first experiment, S. maltophilia K279a was grown overnight at 37°C in LB to prepare fresh 150-mL LB cultures with an initial OD_550_ of 0.05. Cultures were grown to an OD_550_ of 0.2 (~1.5 h, beginning of the exponential phase) and supplemented with the corresponding QS autoinducers as follows: the AHL condition consisted of a combination of C_8_-HSL, 3OC_8_-HSL, and C_10_-HSL at 10 μM each, DSF was added at a final concentration of 10 μM, and the remaining flasks were supplemented with the same volume of DMSO to be used as the control. Cultures were then grown until they reached an OD_550_ of 1.5 (~3 additional hours, transitioning from exponential to stationary growth phase) and harvested for RNA isolation. For the comparison of exponential phase (LOG) and stationary phase (STAT), RNA from triplicate cultures grown in LB was extracted at OD_550_ values of 1.5 and 5, respectively. As for the comparison of K279a WT versus K279aΔ*smlt2053*, starting cultures and extraction time were identical to those of the previous experiment (collected at OD_550_ of 1.5). In all experiments, total RNA was purified with the RNeasy minikit (Qiagen), following the manufacturer’s instructions. The quantity and quality of each RNA sample were assessed with the 2100 Bioanalyzer (Agilent Technologies). Only samples with an RNA integrity number (RIN) of >8 were accepted and selected for library construction.

### RNA library construction and sequencing.

The enrichment of bacterial mRNA was performed by depletion of the bacterial rRNA from total RNA extracted from S. maltophilia cultures. The rRNA was removed using the Ribo-Zero bacteria kit (Illumina) or MICROBExpress bacterial mRNA enrichment kit (Thermo Fisher Scientific) starting with 500 ng or 100 ng, respectively, of bacterial total RNA. The RNA-seq libraries were prepared following the TruSeq stranded mRNA library prep (Illumina) protocol modified by discarding the poly(A) enrichment step. In brief, the enriched bacterial mRNA fraction was fragmented by divalent metal cations at elevated temperature. In order to achieve the library directionality, the second-strand cDNA synthesis was performed in the presence of dUTP. The blunt-ended double-stranded cDNA was 3′ adenylated, and Illumina platform-compatible adaptors with indexes were ligated. The ligation product was enriched with 15 PCR cycles, and the final library was validated on an Agilent 2100 Bioanalyzer (Agilent Technologies) using the DNA 7500 kit (Agilent Technologies).

The libraries were sequenced on HiSeq 2500 (Illumina) or HiSeq 4000 (Illumina) in paired-end mode with a read length of 2 × 76 bp, using TruSeq sequencing by synthesis (SBS) kit v4 (Illumina) or HiSeq 4000 SBS kit (Illumina), respectively, and following the manufacturer’s protocol. Images analysis, base calling, and quality scoring of the run were processed using the manufacturer’s software Real-Time Analysis (RTA 1.18.66.3 or RTA 2.7.7, respectively), followed by generation of FASTQ sequence files.

### RNA-seq data and sequence analysis.

FastQC was used to verify the read quality of the RNA-seq libraries, and reads were mapped against the K279a genome using the STAR aligner. Gene abundances were normalized by calculating fragments per kilobase million (FPKM). The differential gene expression analysis was performed using the DESeq2 R package using an adjusted *P* value of <0.05 (FDR of 5%) as a cutoff for statistical significance, with the Benjamini-Hochberg correction for multiple testing (60). To remove unwanted variation, the surrogate variable analysis (SVA) method (61) was used. For subsequent analyses, loci with fold change of ≥2.0 were assigned as differentially expressed genes (DEGs). The raw, demultiplexed reads as well as coverage files have been deposited in the NCBI’s Gene Expression Omnibus under the accession numbers GSE206442 and GSE206554.

The S. maltophilia K279a proteome was functionally annotated using the eggNOG-mapper (http://eggnogdb.embl.de/#/app/emapper) to assign each protein to a cluster of orthologous groups (COG) category (62). COG categories for the K279a genome were obtained via the Integrated Microbial Genome (IMG) database (https://img.jgi.doe.gov/). The enrichment analyses consisted of a pathway and functional annotation of all DEGs with the topGO R package (v2.38.1) and KOBAS software (v3.0) for the GO and KEGG analysis, respectively (63, 64). Inverted repeats in nucleotide sequences were identified with the palindrome program from the EMBOSS suite (https://www.bioinformatics.nl/cgi-bin/emboss/palindrome). The core genome of 60 genetically diverse strains of S. maltophilia (15) was used to determine gene conservation.

### Quantitative real-time PCR analysis.

Gene expression and analysis were performed to determine the expression ratios of *smlt0264*, *smlt2944*, and *smlt2052* in S. maltophilia K279a wild-type and mutated strain Δ*smlt2053* using specific primers (Table 4). Total RNA was isolated in three different experiments under the same conditions as for the RNA-seq experiments. One microgram of RNA was used to synthesize the cDNA with the Maxima reverse transcriptase kit (Thermo Scientific). qRT-PCR was performed with the CFX96 real-time PCR system (Bio-Rad), and the PCR runs were analyzed with the Bio-Rad CFX Manager software v3.0. Melting curves were done and analyzed for primer quality control. PCR products of ca. 150 bp were amplified for each gene, and *rpoD* was used as a housekeeping control to normalize the gene expression levels. Expression was determined by the threshold cycle (2^−ΔΔ^*^CT^*) method (65).

**TABLE 4:** Primers used in this study

Primer	Sequence^*a*^ (5′–3′)	Application
US′-smlt2053-U	GTTgaattcATTACTCTCAGCGCGAAAC	Upstream flanking region, forward primer to create pΔ*smlt2053*-US′DS′, EcoRI
US′-smlt2053-L	ATGATgctagcCTTGGTCGAGAAGTG	Upstream flanking region, reverse primer to create pΔ*smlt2053*-US′DS′, NheI
DS′-smlt2053-U	CCGCTgctagcTGATTCCACCCAGTAACTG	Downstream flanking region, forward primer to create pΔ*smlt2053*-US′DS′, NheI
DS′-smlt2053-L	TAGagatctCGGTAGTCCTGCTTCTCCA	Downstream flanking region, reverse primer to create pΔ*smlt2053*-US′DS′, BglII
Smlt2053_Protein_U	ATTcatatgGCCAAGCCCGCCCACTTC	Forward primer for cloning *Smlt2053* into pET28b, NdeI
Smlt2053_Protein_L	TCAaagcttTCAAAGGGAAGCGGGCAG	Reverse primer for cloning *Smlt2053* into pET28b, HindIII
US′-smlt2053-2051-U	ACGgaattcCTCTCAGCGCGAAACGA	Upstream flanking region, forward primer to create pΔ*smlt2053-2051*-US′DS′, EcoRI
US′-smlt2053-2051-L	CGAgctagcGGTCCTTGGTCGAGAAGTG	Upstream flanking region, reverse primer to create pΔ*smlt2053-2051*-US′DS′, NheI
DS′-smlt2053-2051-U	TCGgctagcTGTAAGGATCAAGGCGTTG	Downstream flanking region, forward primer to create pΔ*smlt2053-2051*-US′DS′, NheI
DS′-smlt2053-2051-L	TGAagatctGCAGCTGCTGAAGACCGAAGA	Downstream flanking region, reverse primer to create pΔ*smlt2053-2051*-US′DS′, BglII
Ext-smlt2053-2051-U	TCGACGAAGAACTTCTCCA	Forward primer outside the deleted region for mutant verification
Ext-smlt2053-2051-L	GATCTTCGAGCTGCTGGAG	Reverse primer outside the deleted region for mutant verification
Smlt2053_Comp_U	CATtctagaATAAACGTAGGCGAATTG	Forward primer for cloning gene *Smlt2053* into pBBR1MCS-1 including its own promoter, XbaI
Smlt2053_Comp_L	TTTaagcttGCAGTTACTGGGTGGAATC	Reverse primer for cloning gene *Smlt2053* into pBBR1MCS-1, HindIII
rt_smlt0077_U	AGCCGTGGGACACCTATAC	Gene expression by qRT-PCR
rt_smlt0077_L	ACTTCGTTGAGCTGCTCG	Gene expression by qRT-PCR
rt_smlt2944_U	TCTCCAACTTCAACATCCA	Gene expression by qRT-PCR
rt_smlt2944_L	ATTGCCGAGCTGTCCATA	Gene expression by qRT-PCR
rt_smlt2053_U	CAAGTTCCTGTCCGACCA	Gene expression by qRT-PCR
rt_smlt2053_L	CTTGATCAGCCCGAAGTC	Gene expression by qRT-PCR
rt_smlt0264_U	GAGAACATGAGCGAGCTGG	Gene expression by qRT-PCR
rt_smlt0264_L	TAGGTGTCCACGCCATTG	Gene expression by qRT-PCR
rt_smlt2051_U	AGCGGTTCACGGTCTGTGCGG	Gene expression by qRT-PCR
rt_smlt2051_L	CCCGCCCCGACGACATGCTTG	Gene expression by qRT-PCR
rt_smlt2052_U	CGCACGGCCAGTTCCTTCAGG	Gene expression by qRT-PCR
rt_smlt2052_L	ACTTCCAGCGCACCAGCCAGC	Gene expression by qRT-PCR
rt_smlt1574_U	GCCGCGGATGTGGTTGAACAG	Gene expression by qRT-PCR
rt_smlt1574_L	TGATGGAGGAAGCGGGCTACC	Gene expression by qRT-PCR
rt_smlt2180_U	GCACCGTTCGGCCTGATCCAG	Gene expression by qRT-PCR
rt_smlt2180_L	CGTTGCCGGTATCGAAATCGC	Gene expression by qRT-PCR
rt_rpoD_U	AAGGGCCTGGAACAGGGCTA	Housekeeping for qRT-PCR^*b*^
rt_rpoD_L	CCACATCCGGCGCAACTTCA	Housekeeping for qRT-PCR^*b*^
smlt2053_EMSA_Up	GGGATGTCTCCAATGGAATC	DNA probe used in EMSA (amplicon size of 312 bp)
smlt2053_EMSA_Low	GGTGGGCGGACCTTAATC	DNA probe used in EMSA (amplicon size of 312 bp)
Smlt0077_EMSA_Up	ATTGAATTCAGGAAGCGAGAGAAGTGC	DNA probe used in EMSA (amplicon size of 610 bp)
Smlt0077_EMSA_Low	ATAGCTAGCGGTATAGGTGTCCCACGG	DNA probe used in EMSA (amplicon size of 610 bp)
Smlt2944_EMSA_Up	CGCTTGCGCATTGTCTGT	DNA probe used in EMSA (amplicon size of 435 bp)
smlt2944_EMSA_Low	TGCATCCTCCCCTGACGA	DNA probe used in EMSA (amplicon size of 435 bp)

a Restriction site is shown in lowercase letters.

b Primers have been described previously (58).

### Construction of unmarked deletion mutants.

Markerless S. maltophilia K279a mutants were constructed according to the method initially developed for multidrug-resistant B. cenocepacia, using the pGPI-SceI/pDAI-SceI-SacB system (66). Briefly, this method is based on the I-SceI homing endonuclease system, which relies on two independent crossover events to generate markerless deletion mutants. First, the suicide plasmid pGPI-SceI-XCm (58, 67) containing an I-SceI recognition site and the flanking regions of the target region to be deleted (see Tables 3 and 4 for plasmid construction and primer details, respectively) is introduced into the wild-type strain of S. maltophilia by triparental mating. After this first crossover event, the second plasmid pDAI-SceI-SacB is introduced. This second plasmid expresses the I-SceI endonuclease, which resolves the cointegrate structure with a second recombination event, stimulating the DNA repair machinery of the host strain to produce the markerless mutants. The pDAI-SceI-SacB plasmid is cured by sucrose counterselection. Primers external to the deleted region (Table 4) were used to verify the mutants by both PCR and Sanger sequencing.

### Protein production and purification.

To obtain the purified protein Smlt2053, the entire coding sequence was cloned, along with its upstream promoter region, into expression plasmid pET28a-TEV (primers listed in Table 4), which was electroporated into Escherichia coli BL21(DE) as the expression host system. Immobilized-metal affinity chromatography (IMAC) purification was used to obtain the recombinant protein. Briefly, the resulting pellet of a 2-L overnight culture was resuspended in 5 mM imidazole, 300 mM NaCl, and 20 mM Tris-HCl, pH 7.9. After three rounds of sonication, the sample was centrifuged for 45 min at 15,000 × *g* to separate the soluble and insoluble fractions. The soluble fraction was purified in a His-Trap (GE) column prior to the gradient elution in an ÄKTA fast-performance liquid chromatography (FPLC) pure platform. All the expression and purification procedures were performed by the Protein Production Platform (PPP) from Nanbiosis facilities at the Institute of Biotechnology and Biomedicine (IBB) from the Universitat Autònoma de Barcelona (UAB), Barcelona, Spain. Protein identity was confirmed by peptide mass fingerprinting (matrix-assisted laser desorption ionization–time of flight mass spectrometry [MALDI-TOF MS]) at the Proteomics Laboratory from the Consejo Superior de Investigaciones Científicas (CSIC) and UAB.

### EMSAs.

EMSAs were adapted from the protocol of Hellman and Fried (68). The DNA fragment containing the promoter region of the target gene was generated by PCR using specific primers (Table 4). Binding reactions were carried out in a Tris-glycine buffer (10 mM to 40 mM) containing 20 mM KCl, 7.5% glycerol, 0.01 mg/mL bovine serum albumin (BSA), and 1 mM dithiothreitol (DTT). Reaction mixtures consist of 50 ng of the DNA probe, 0 to 300 ng of purified protein Smlt2053, and 20 ng/μL poly(dI·dC) in a final volume of 20 μL. When required, different-length fatty acids or DSF were included in the binding reaction at various concentrations that ranged from 1 μM to 1,000 μM. The same solvent used to dissolve each fatty acid was used as a control in each assay. When the reaction mixture included a testing compound, the protein Smlt2053 was used at a fixed concentration of 429 nM (11.25 ng/μL), and the DNA-protein binding was incubated for 20 min before addition of the compound.

Reaction mixtures were incubated for 30 min at 4°C and separated by electrophoresis on a native 6% polyacrylamide gel (acrylamide/Bis-acrylamide, 37.5:1) in 0.5× Tris-borate-EDTA (TBE) buffer (pH 8.3) containing 2.5% glycerol at 4°C in a cold room. After electrophoresis, gels were rinsed with water and stained with a 0.5× TBE solution containing 1× RedSafe (Sigma-Aldrich) for 15 min. To remove the exceeding dye, gels were washed twice with water for 10 min. Gels were visualized in a VersaDoc imaging system (Bio-Rad).

### DSF extraction, detection, and quantification.

Crude DSF was extracted from culture supernatants with the ethyl acetate method (48). Bacteria were removed by centrifugation from 200-mL culture aliquots, and the supernatants were extracted three times with 1/3 of the volume of ethyl acetate. The combined extracts were evaporated to dryness in a rotary evaporator, dissolved in 3 to 4 mL ethyl acetate, and evaporated again in the SpeedVac. Residues were dissolved in 200 μL of 30% methanol. DSF determination was performed using the reporter strain X. campestris pv. *campestris* 8523 pL6engGUS as previously described (11). Blue halo zone widths in the bioassay plate were measured, and DSF quantification was done by correlating these values to a dose-response plot of the biosensor strain to DSF. The quantitative experiment was done in triplicate with a correlation coefficient (*r*^2^) of the regression curves of 0.93 to 0.99.

### Biofilm formation.

For biofilm production, cells were grown in BM2 glucose minimal medium supplemented with Casamino Acids in 96-well microtiter plates, and biofilm quantification was performed by the crystal violet assay as previously described (7) with modifications. Overnight cultures were used to inoculate 200 μL of growth medium in each well of the 96-well plate (initial OD_550_ of 0.05). The plates were incubated in static conditions at 37°C for 20 h for biofilm growth. After incubation, wells were washed three times, fixed at 60°C for 1 h, and stained for 15 min with a crystal violet solution at 0.1%. The stained biofilms were rinsed with distilled water, allowed to dry at 37°C for 30 min, and then extracted with 200 μL of 30% acetic acid. The amount of biofilm was quantified by measuring the OD_550_ of dissolved dye using the Multiskan FC reader. These experiments were carried out at least with triplicate samples.

### Analysis of cellular fatty acids.

Cellular fatty acid analysis was done at the Spanish Type Culture Collection (CECT; University of Valencia, Spain). Cells were grown in LB medium for 24 h at 37°C; extractions and determinations were performed according to the standard protocol of the MIDI Microbial Identification System (MIS; Microbial ID Inc., Newark, DE, USA) (69) using an Agilent 6850 gas chromatograph (Agilent Technologies) following the Sherlock TSBA6 method and library.

### Statistical analysis.

Two-way analysis of variance (ANOVA) with Tukey’s multiple-comparison test was used to determine the significance of the data between groups. All statistical analyses were performed by GraphPad Prism software (v 5.0; GraphPad Inc, San Diego, USA), considering a *P* value of less than 0.05 as statistically significant.

### Data availability.

A full list of significant DEGs from each experimental condition is provided as a data supplement, and all raw sequence data are available at the NCBI’s Gene Expression Omnibus under the accession numbers GSE206442 and GSE206554.
