# Supplementary material for: A Stenotrophomonas maltophilia TetR-Like Transcriptional Regulator Involved in Fatty Acid Metabolism Is Controlled by Quorum Sensing Signals
Source: Appl Environ Microbiol. 2023 Jun 5;89(6):e00635-23. doi: 10.1128/aem.00635-23 (PMC10304680; doi:10.1128/aem.00635-23)
Supplement: Supplemental file 1 — Supplemental material. Download aem.00635-23-s0001.pdf, PDF file, 1.6 MB [file aem.00635-23-s0001.pdf]

A *Stenotrophomonas maltophilia* TetR-like transcriptional regulator involved in fatty acid metabolism is controlled by quorum sensing signals.

Xavier Coves[1,2,§], Marc Bravo[1,2,§], Pol Huedo[1,§], Òscar Conchillo-Solé[1,2], Andromeda-Celeste Gómez[1,2], Anna Esteve-Codina[3,4], Marc Dabad[3], Marta Gut[3], Xavier Daura[1,5,6], Daniel Yero[1,2] and Isidre Gibert[1,2]

[1]Institut de Biotecnologia i de Biomedicina (IBB), Universitat Autònoma de Barcelona (UAB), 08193 Cerdanyola del Vallès, Spain. [2]Departament de Genètica i de Microbiologia, Universitat Autònoma de Barcelona (UAB), 08193 Cerdanyola del Vallès, Spain. [3]CNAG-CRG, Centre for Genomic Regulation (CRG), The Barcelona Institute of Science and Technology, 08028 Barcelona, Spain. [4]Universitat Pompeu Fabra (UPF), Barcelona, Spain. [5]Catalan Institution for Research and Advanced Studies (ICREA), 08010 Barcelona, Spain. [6]Centro de Investigación Biomédica en Red de Bioingeniería, Biomateriales y Nanomedicina, Instituto de Salud Carlos III, 08193 Cerdanyola del Vallès, Spain.

[§] These authors have contributed equally to this work.

* Co-corresponding authors: Daniel Yero, Daniel.Yero@uab.cat and Isidre Gibert, Isidre.Gibert@uab.cat.

Supplementary Figures

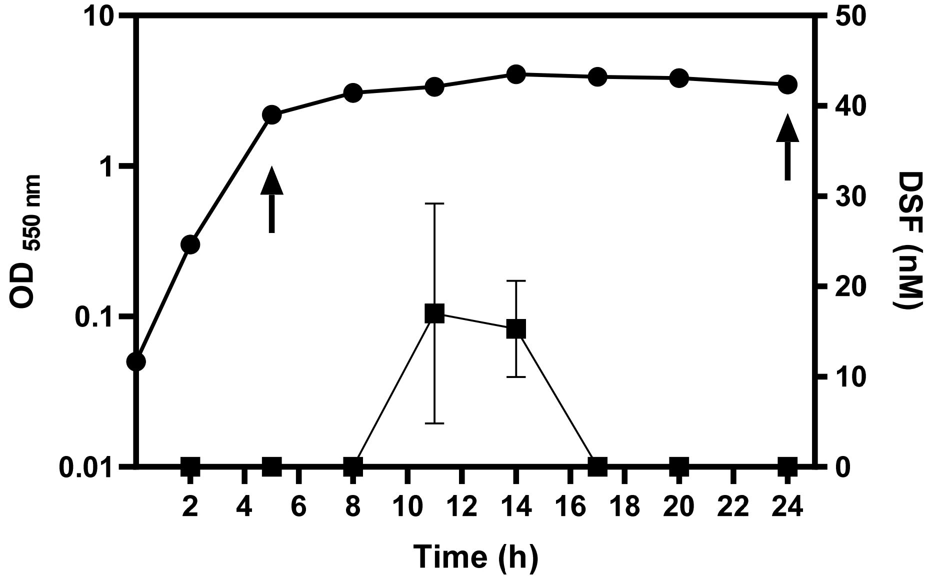

Figure S1. Time course of DSF production (closed box) during growth of *Stenotrophomonas maltophilia* K279a (closed circle). Cells of an overnight culture of K279a were diluted to OD_{550nm} of 0.05 in fresh LB medium and cultivated at 37°C. Samples were taken at the times indicated and autoinducer concentrations in the medium were determined based on a bioassay the DSF reporter *Xanthomonas campestris* 8523 pL6engGUS. The error bars are the means ± SD (n = 3). Arrows indicate time points at which RNA samples were collected to compare global gene expression between the exponential and stationary growth phase.

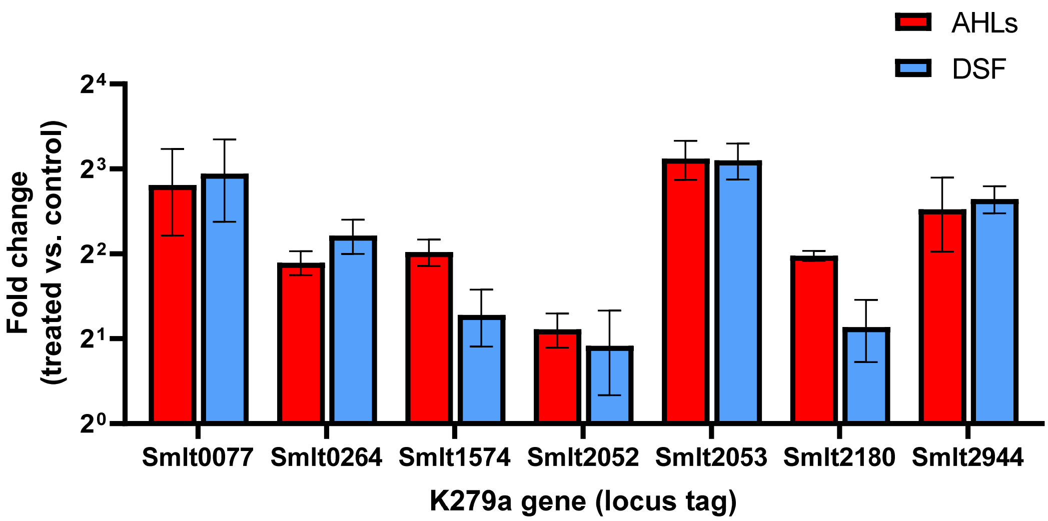

Figure S2. qRT-PCR validation of gene expression observed in RNA-Seq data for differentially expressed representative genes after supplementation with either QS autoinducers AHLs or DSF. The Y-axis indicates relative normalized expression. Expression folds are expressed relative to the DMSO-treated group and RNA polymerase sigma factor *rpoD* gene was used as the normalizing gene. The error bars are the means ± SD (n = 3).

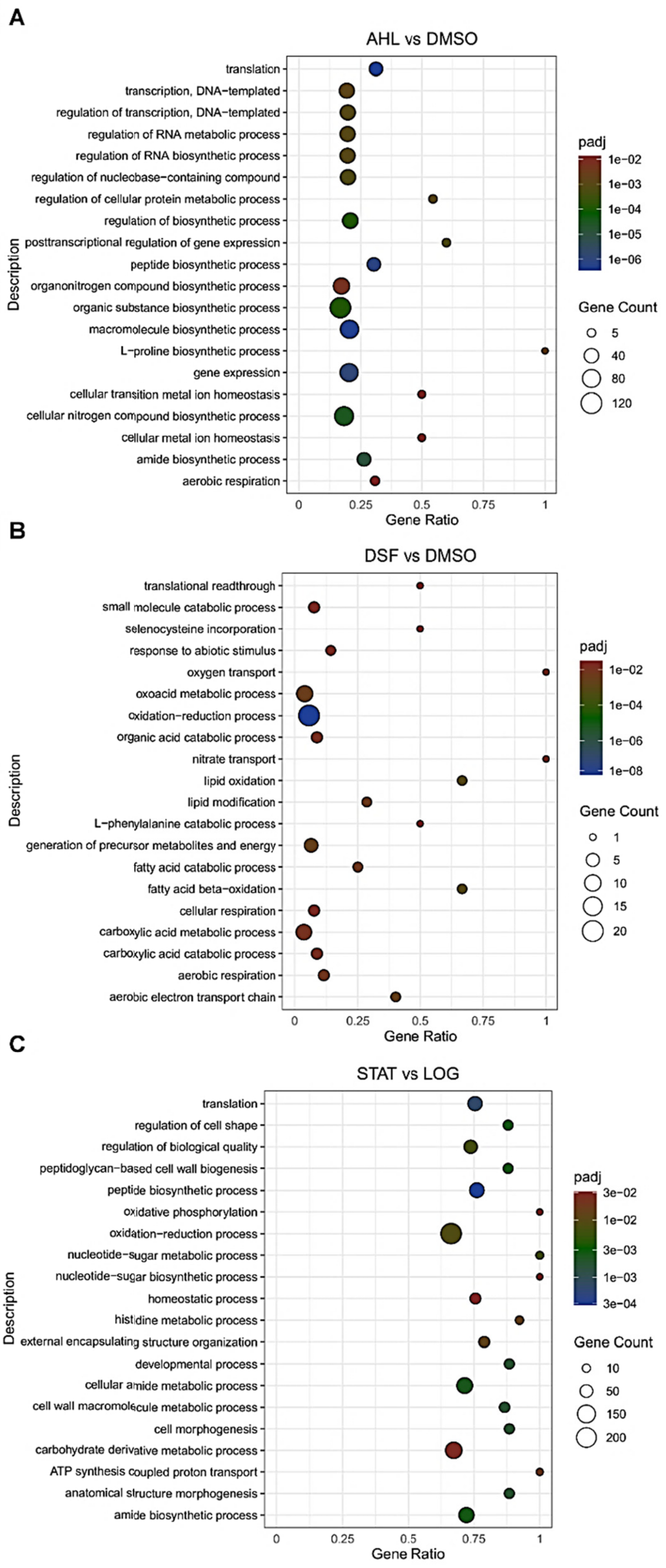

Figure S3. Top 20 GO terms enriched in each condition.

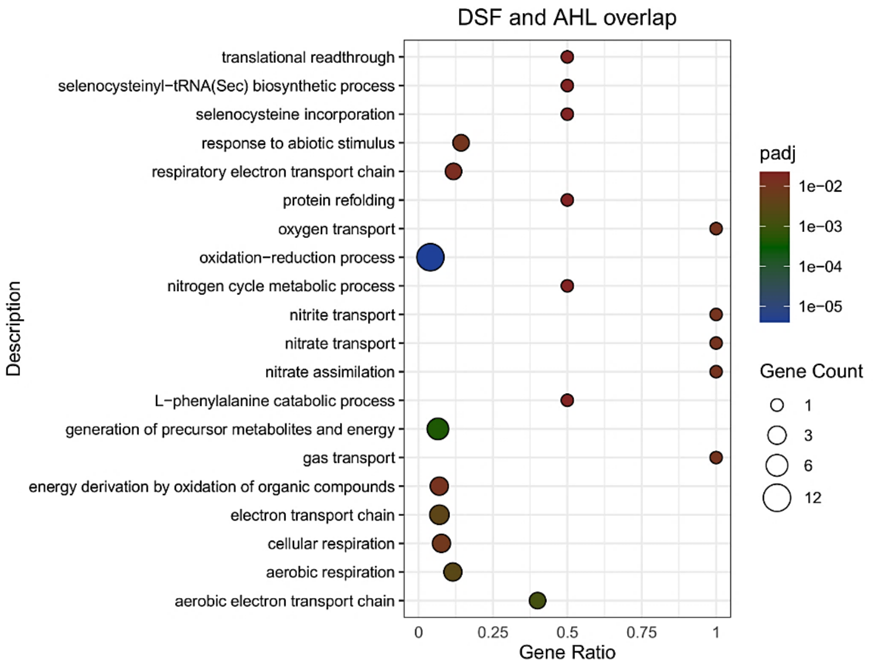

Figure S4. Top 20 GO terms in the DEGs common in the AHL and DSF conditions.

Figure S3. Top 20 GO enriched terms in each condition. Y axis indicates the corresponding GO term, while X axis indicates de gene ratio (number of terms in our dataset with respect to the total number of annotations in the QuickGO database for this strain). The number of genes matching each category in our dataset is represented by the Size of the bubbles and the color of the bubble indicates the significance of each term (p-value with Fisher's hypergeometric test correction).

AHL: Supplementation with a combo of C8-HSL, 3OC8-HSL and C10-HSL (10 µM each). DSF: Supplementation with 10 µM DSF. DMSO: Control for AHL and DSF conditions with the same volume of DMSO used to dissolve each signal. STAT: Culture harvested in the stationary phase of growth. LOG: Culture harvested in the mid-late exponential phase of growth.

Figure S4. Top 20 GO terms in the DEGs common in the AHL and DSF conditions. Y axis indicates the corresponding GO term, while X axis indicates de gene ratio (number of terms in our dataset with respect to the total number of annotations in the QuickGO database for this strain). The number of genes matching each category in our dataset is represented by the Size of the bubbles and the color of the bubble indicates the significance of each term (p-value with Fisher's hypergeometric test correction).

A

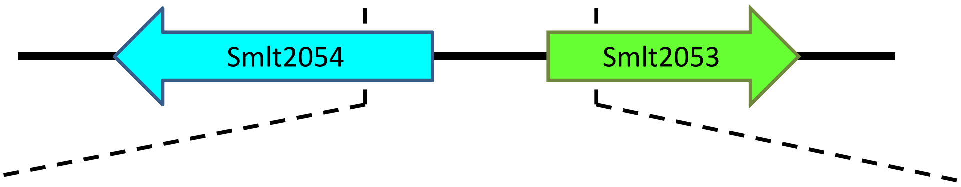


```
GTCGCGGTGGGCCAGGACGGCGTTCTCGCCTTCCAGGGCCTGGATCATCACCGGGCCGGAGATCATGAAC
TCGACCAGCGCGTTGAAGAACGGACGCTCGCGGTGCACGGCGTAGAAGCCTTCGGCTTCACGACGCGACA
GCTGCTTGTACTTGGCGGCCACGACCTTCAGGCCGGCCTTCTCGAAGCGGGCGTAGATTTCGCCGATGAC
GTTCTTGGCAACGGCGTCCGGCTTGATGATCGAAAGGGTGCGCTCCAGCGCCATGGGATGTCTCCAATGG
AATCGGGCCGCTGATGGCCCGAAGGGTAACATGAAAAAAACCCGCGTCGGGACGCGGGCTTAGCCTGATA
AACGTAGGCGAATTGTAAAAGATAAAGCGGCGGCATGGTAGTGCCGGCCGCTGGCCGGCATTGGGCGCCA
TTCAACCTCGGGGCTGCCGGCCAGCGGCCGGCACTACCCCATTCACCCACCTGAGTGAAATCTGACCGGA
TCGGCCGATTTTGCTGCACTGCAAAATGCCATACGGTACCGTCTGGTCCTAGTATCAAACAATCGTTTGA
TTAAGGTCCGCCCACCGATGGCCAAGCCCGCCCACTTCTCGACCAAGGACCGGATCCTCGGCGCAGCCGA
GGAGCTGTTCGCCCAGCACGGCTTCGCTGGCACCTCGCTGCGCCAGGTGACCAGCCAGGCCGACGTCAAC
ATCGCCGCGGTCAACTACCACTTCGGCTCCAAGGAAAACCTGGTCAACGAGGTGTTCCGCCGCCGCATGG
ACGAGATGACCGGTGCCCGCCTGTCGCAGCTCGAGCGCGCCCGCGCCGAGCACCCGGGTGAACTGCGCCC
GGTGCTGGCTGCCTTCGTCGAGCCGGCCCTGGCGCTGGCCCAGGACCGCCAGAACGGCGGCGCCTTCGTG
CGCGTGATCGCCCGCGCCTACGCCGAGAAGAACGACAACCTGCGCAAGTTCCTGTCCGACCACTACGGCC
ACGTGCTGCGTGCGTTCGGCAAGGCCATCGGCGAGTGCGTGCCGGACCTGAGCAAGGAGGAGCTGTACTG
```

B

```
K279a    CAAAATGCCATACGGTACCGTCTGGTCCTAGTATCAAACAATCGTTTGATTAAGGTCCGCCCACC-GATG
D457     CAAAATGCCATACGCTACCGTCTGGTCCTAGTATCAAACAATCGTTTGATTAAGGTCCGCCCGCC-GATG
JV3      CAAAATGCCATACGCTACCGTCTGGTCCTAGTATCAAACAATCGTTTGATTAAGGTCCGCCCGCC-GATG
R551-3   CAAAATGCCATACGCTACCGTCTGGTTCTAGTATCAAACAATCGTTTGATTAAGGTCCGCCTGCCCGATG
         ************** *********** ************************************   ** ****
```

Figure S5. (A) Intergenic DNA sequence between genes Smlt2054 (blue) and Smlt2053 (green) in the K279a genome (NC_010943.1). In the promoter $P_{Smlt2053}$ two palindromic sequences are highlighted: the canonical sequence G/CAAAC(N_{2-4})GTTTG/C found in other ortolog TetR-like regulators in yellow and a more divergent palindrome in brown. The DNA segment amplified by PCR to be used as a probe in the EMSA is underlined. (B) Multiple alignment (done with ClustalW) showing the conservation of the promoter region containing the consensus sequences among the strains of *S. maltophilia* K279a, D457 (NC_017671.1), JV3 (NC_015947.1) and R551-3 (NC_011071.1).

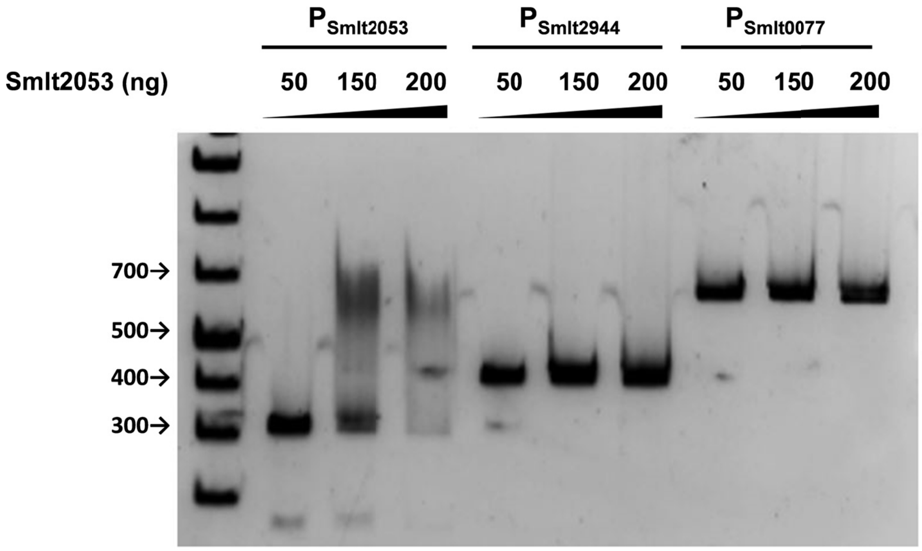

Figure S6. EMSA experiment showing that protein Smlt2053 does not bind to DNA probes containing promoter $P_{Smlt2944}$ or $P_{Smlt0077}$ at a fixed concentration of 50 ng each. DNA probe containing promoter $P_{Smlt2053}$ was also included. 1 Kb Plus DNA ladder (Thermo Fisher Scientific) was used as molecular weight marker.

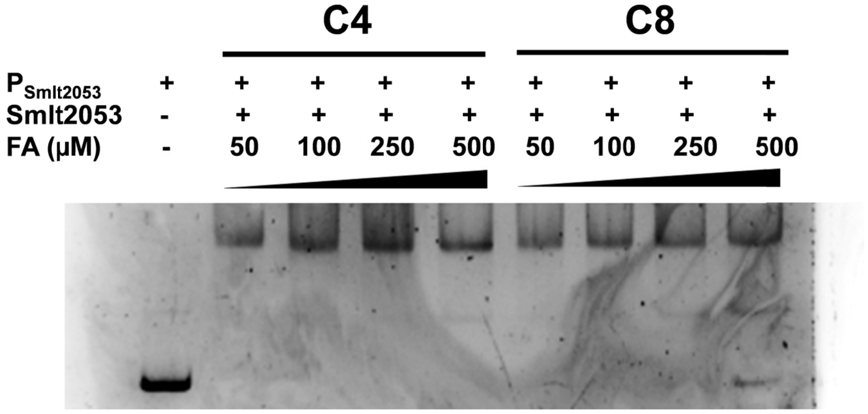

Figure S7. Medium or long chain fatty acids do not affect the binding of the Smlt2053 protein to the DNA probe containing its own promoter region. EMSA with different amount of each fatty acid, protein Smlt2053 at a fixed concentration of 429 nM and the DNA probe containing promoter $P_{Smlt2053}$ at 50 ng.

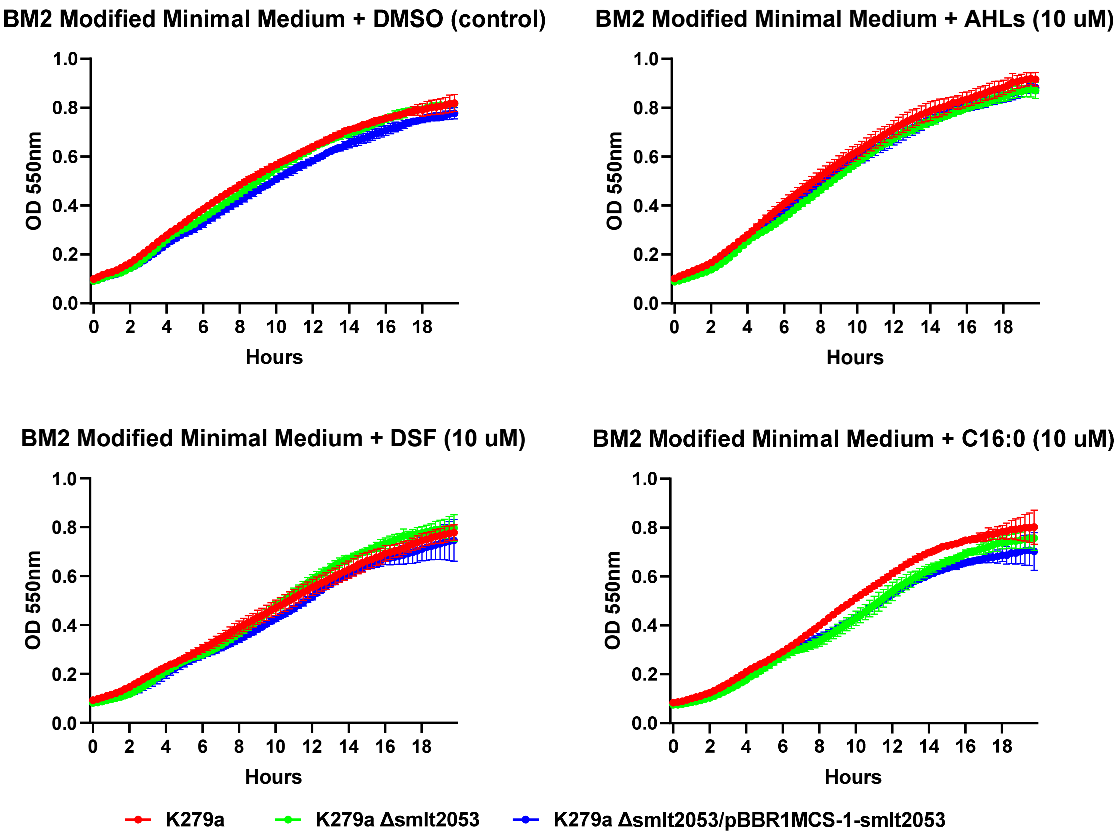

Figure S8. Growth curves of *S. maltophilia* K279a, the derivative mutant strain K279a Δ*smlt2053* and the complemented strain carrying the plasmid pBBR1MCS-1-*smlt2053*. Bacterial cells were grown on BM2 minimal medium with glucose (0.4%) and casamino acids (0.5%) at 37°C with continuous shaking. OD 550nm, optical density at 550 nm.

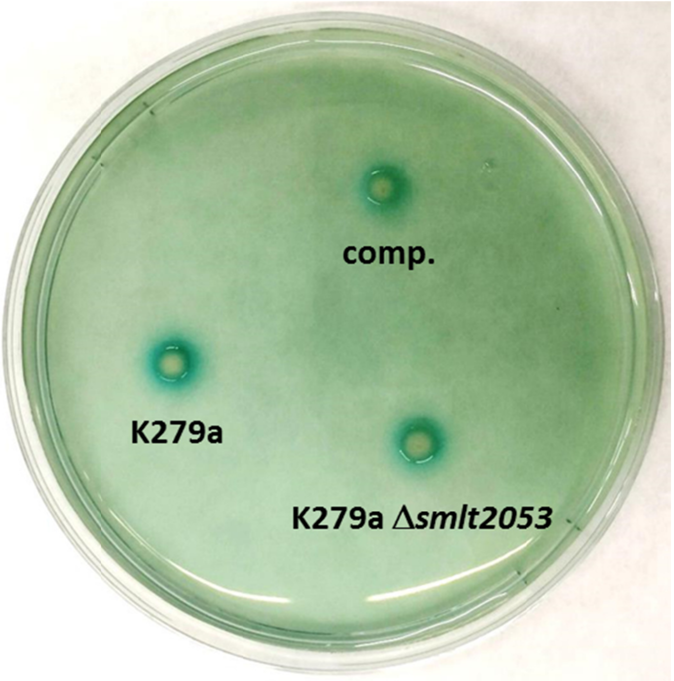

Figure S9. Colony DSF bioassay of *S. maltophilia* K279a, its isogenic mutant strain K279aΔ*smlt2053* and the complemented (comp.) strain K279aΔ*smlt2053*/pBBR1MCS-1-*smlt2053*. A modification of the bioassay for the detection of DSF was done. The three strains were grown overnight in BM2 minimal medium with glucose (0.4%) and casamino acids (0.5%) and pin inoculated onto plates of BM2 agar containing casamino acids (0.5%), glycerol (2%) and X-Glu (80 μg/ml) and seeded with the DSF reporter strain.
